# Association between CCL5, CCL11, and CCL17 polymorphisms and atopic dermatitis risk: A systematic review and meta-analysis

**DOI:** 10.1097/MD.0000000000036897

**Published:** 2024-02-23

**Authors:** Chenghui Zou, Wen Zhang, Mao Li, Dan He, Yujie Han, Min Liu, Mao Lu

**Affiliations:** a School of Clinical Medicine, Chengdu Medical College, Chengdu, Sichuan, China; b Department of Dermatovenereology, The First Affiliated Hospital of Chengdu Medical College, Chengdu, Sichuan, China.

**Keywords:** atopic dermatitis, chemokines, meta-analysis, polymorphism

## Abstract

**Background:**

Atopic dermatitis (AD) is a common and recurrent inflammatory disease with strong genetic susceptibility. The abnormal production of chemokines plays an important role in the occurrence and development of AD.

**Methods:**

A comprehensive online literature search was performed in databases of China National Knowledge Infrastructure, Wanfang, VIP China Science and Technology Journal Database, China Biomedical Literature Database, PubMed, Embase and Cochrane Library to retrieve relevant articles published from January 2000 to October 2022. The odds ratio (OR) with its 95% confidence interval (CI) was employed to calculate this relationship.

**Results:**

A total of 7 studies were finally screened out, including 1316 AD patients and 1099 controls. There were 3 studies for CC chemokine ligand 5 (CCL5) polymorphisms, 2 for CCL11 polymorphisms, and 2 for CCL17 polymorphisms, respectively. The meta-analysis revealed a significant association between the CCL5 − 403G/A polymorphism and AD under the allelic model (A vs G: OR = 1.25, 95% CI = 1.02–1.52, *P = *.03), heterozygous model (AG vs GG: OR = 1.40, 95% CI = 1.08–1.80, *P = *.01) and dominant model (AA + AG vs GG: OR = 1.38, 95% CI = 1.08–1.76, *P = *.01) in a fixed-effect model. The allelic model (G vs C: OR = 1.46, 95% CI = 1.07–1.98, *P < *.01) and dominant model (GG + GC vs CC: OR = 1.74, 95% CI = 1.23–2.47, *P < *.001) of the CCL5 − 28C/G polymorphism were also associated with an increased risk of AD. However, this significant association was not found in other alleles and genotypes (*P > *.05).

**Conclusion:**

Our results show that the A allele, AG and AA + AG genotypes of the CCL5 − 403G/A polymorphism, the G allele and GG + GC genotype of the CCL5 − 28C/G polymorphism are risk factors for AD. Future studies with large population are still needed to further explore those correlations.

## 1. Introduction

Atopic dermatitis (AD) is a common chronic and recurrent inflammatory skin disease with high heterogeneity and heredity.^[1]^ It is characterized by dryness, recurrence, pruritus and eczematous skin lesions,^[2]^ and it has caused a huge burden on medical resources.^[3]^ AD usually starts in infancy or early childhood and can affect 15% to 30% of children and up to 10% of adults.^[4]^ More than 60% of children with AD suffer from asthma and allergic rhinitis.^[5]^ The prevalence of AD varies in different regions around the world.^[6]^ In the United States, it affects about 17.8 million people.^[7]^ Although the exact cause of AD is still unclear, genetic susceptibilities such as interleukin (IL) type, chemokines and environmental factors such as microorganisms (*Malassezia*), play an important role in the occurrence and development of AD.^[8]^ Many AD patients can obtain satisfactory effects through the external use of drugs and antipruritics, however, some patients still need more active systematic treatment.^[9]^ Therefore, the exploration of new biomarkers can better understand the pathogenesis of AD, so as to find the disease early and carry out targeted treatment.

AD is mediated by type 2 T helper (Th2) cells. Specific immune and inflammatory mechanisms play an important role in the development of AD, and the abnormal production of cytokines and chemokines is related to its pathogenesis.^[10]^ Chemokines are a group of cytokines that help cells migrate, grow, differentiate and regulate the inflammatory and anti-inflammatory responses of the immune system.^[11]^ Chemokines are the most potent molecules regulating the selective recruitment of leukocytes into inflamed lesions.^[12,13]^ In addition, some chemokines also affect IL production, angiogenesis and collagen production.^[12,14]^ Based on a cysteine motif, CXC, CC, C and CX3C families have been identified.^[15]^ CC (or *β*) chemokines exert their action on multiple leukocyte subsets, including monocytes, basophils, T cells, dendritic cells (DCs) and natural killer cells.^[12]^ They are also associated with cancer and inflammatory diseases. CC chemokine ligand 5 (CCL5) is located in the human chromosome 17q11.2–q12.16 region.^[16]^ CCL5 can recruit chemotactic T cells, eosinophils and basophils to inflammatory sites. CCL11, located in the human chromosome 17q21.1–q21.2 region,^[17]^ is composed of 3 exons. As an effective chemokine and activator of eosinophils and helper T cells, it is also associated with the pathogenesis of AD. CCL17 is located in the human chromosome region 16q13. It is related to the pathogenesis of Th2-based allergic diseases such as bronchial asthma and AD.^[18]^ Other studies have also proved that serum CCL17 protein levels in patients with bronchial asthma and AD are significantly higher than those in healthy individuals.^[19,20]^

The function of CC chemokines genes may affect the composition and induction pathways of cytokines, leading to an imbalance of chemokine-related factors. This imbalance may in turn alter the susceptibility to AD. Many studies have identified the role of CC chemokines genes in AD,^[21,22]^ however, the results remain inconclusive. Therefore, we conducted this meta-analysis to systematically review all the published studies on this issue to obtain a relatively reliable results of CCL5, CCL11 and CCL17 polymorphisms in AD susceptibility.

## 2. Methods

### 2.1. Strategies of literature search

A comprehensive online literature search was performed in databases of China National Knowledge Infrastructure, Wanfang, VIP China Science and Technology Journal Database, China Biomedical Literature Database, PubMed, Embase and Cochrane Library to retrieve relevant articles published from January 2000 to October 2022. The following terms: “atopic dermatitis or atopic eczema or atopic diseases” and “chemokines genes or CC genes or CCL5 or CCL11 or CCL17” as well as their combination and synonyms were employed as the searching words. The references of related studies were manually searched to obtain more studies. The search was only limited to English and Chinese languages. When the same authors or laboratory reported the same issue on the same population, only the recent full text was included. Details of the search procedure and strategy were presented in Appendix.

### 2.2. Inclusion and exclusion criteria

The eligible studies must meet the following criteria: a case-control study focused on the role of CCL5, CCL11 and CCL17 polymorphisms in AD risk; patients should meet the diagnostic criteria of AD described by Hanifin and Rajka,^[23]^ controls should be age- and sex-matched healthy participants; the allele and genotype information of patients and controls were available to extract; the results were presented in odds ratio (OR) with its 95% confidence interval (CI). The exclusion criteria were: studies with duplicate data; without a control group; review or conference papers; (data could not be extracted.

### 2.3. Data extraction

According to the Preferred Reporting Items for Systematic Reviews and Meta-Analyses guidelines, all data from each included study was extracted and assessed by 2 investigators independently, and any disagreement was discussed with a third expert to achieve a consensus. The following information was extracted: the first author, year of publication, country, ethnicity, mean age, sample size, genotype methods, allele and genotype distributions in AD cases and controls, and Hardy Weinberg Equilibrium in controls.

### 2.4. Quality evaluation

Quality assessment was carried out using the risk of bias tool recommended by the Cochrane Collaboration.

### 2.5. Statistical analysis

The strength of the association between polymorphisms of CCL5, CCL11 and CCL17 and AD risk was measured by the crude ORs with their 95% CIs under 5 genetic models: the allelic model (A vs a), homozygous model (AA vs aa), heterozygous model (Aa vs aa), dominant model (AA + Aa vs aa), and recessive model (AA vs Aa + aa). The significance of the pooled ORs was determined by the Z test with a *P* value < .05 considered statistically significant. Between-study heterogeneity was measured by the *I^2^* test and the Q statistic test. The fixed-effect model was used when the effect was homologous (*I^2^* < 50% and *P* value of the Q test > 0.01). Otherwise, the random-effect model was employed when the effect was heterogenous. All analyses were performed by Review Manager 5.4 (The Cochrane Information Management System).

## 3. Results

### 3.1. Study characteristics

**Figure 1.**
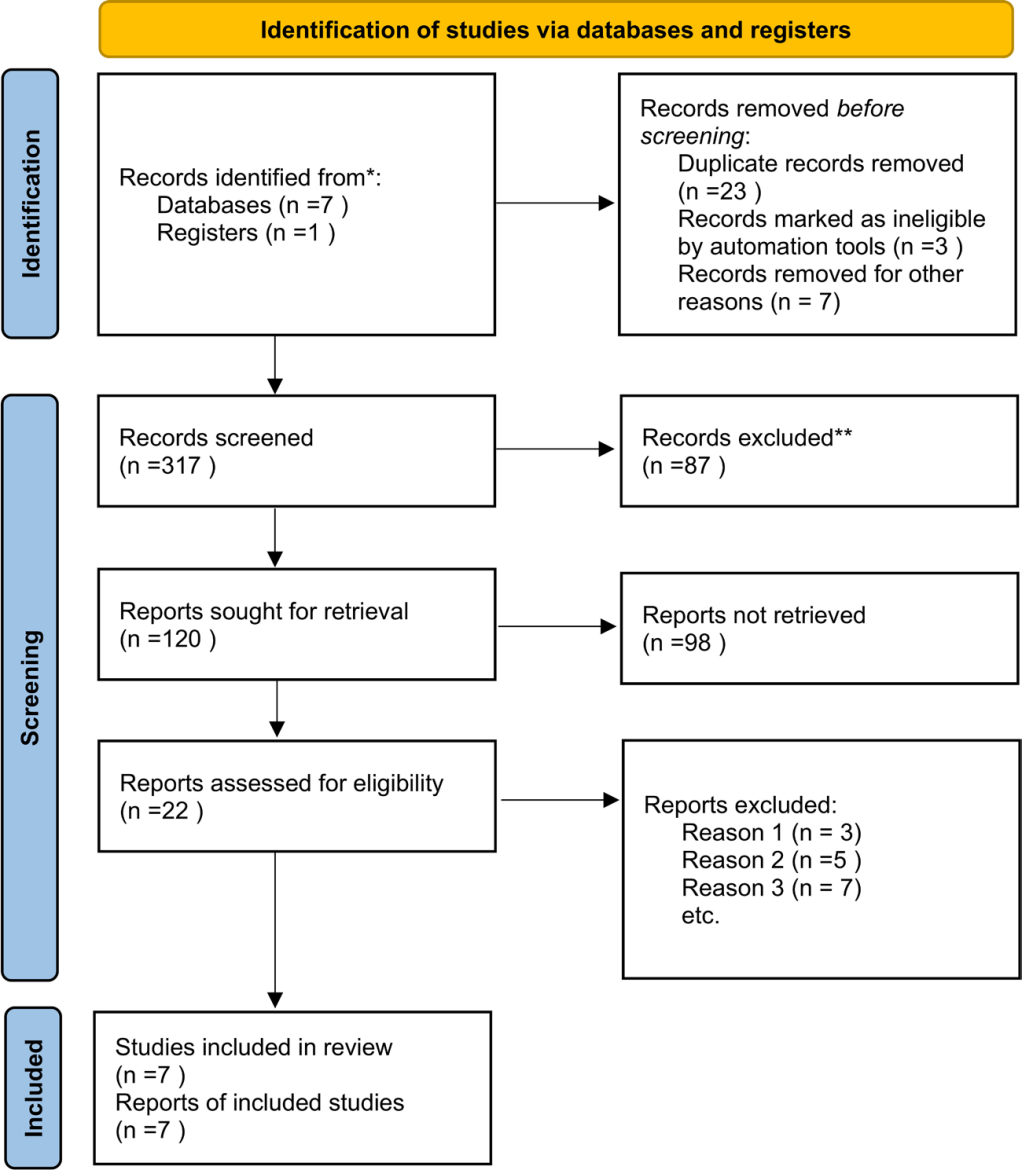
Flow diagram of the study selection process.

After applying the inclusion and exclusion criteria, a total of 7 studies were finally screened out, including 1316 AD patients and 1099 controls. Figure 1 showed the selection process of this meta-analysis. Of the 7 studies, 4 were written in Japan,^[16–18,24]^ 1 in Germany,^[25]^ 1 in Hungary,^[26]^ and 1 in Italy.^[27]^ Three genes containing 6 polymorphic sites were concerned: CXCL5 (−403G/A, −28C/G), CCL11 (−426C/T, −384A/G, −67G/A) and CCL17 (−431C/T). The included studies were conducted in 9 countries containing 2 ethnicities (Asian and Europe). The sample size ranged from 195 to 566. The genotype distributions in the controls of all studies were in agreement with Hardy Weinberg equilibrium except for 1 study for CCL11.^[17]^ The main characteristics of included studies were presented in Table 1. The allele and genotype information were listed in Table 2.

**Table 1:** Main characteristics of included studies in this meta-analysis.

First author	Yr	Country	Ethnicity	Mean age		Sample size		Genotyping	Gene polymorphism
				Cases	Controls	Cases	Controls	method	
Nickel	2000	Germany	Europe	−	−	188	98	PCR-SSCP	CCL5 − 403G/A
Kozma	2002	Hungary	Europe	−	6.3 ± 4.4	128	303	PCR	CCL5 − 403G/A − 28C/G
Tanaka	2006	Japan	Asia	24.7	34.9	389	177	PCR	CCL5 − 403G/A − 28C/G
Rigoli	2007	Italy	Europe	10.5	12.0	130	65	PCR-RFLP	CCL11 − 426C/T − 384A/G − 67G/A
Tsunemi	2002	Japan	Asia	28.0 ± 7.3	−	140	140	PCR-RFLP	CCL11 − 426C/T − 384A/G − 67G/A
Sekiya	2003	Japan	Asia	−	−	148	158	PCR-RFLP	CCL17 − 431C/T
Tsunemi	2004	Japan	Asia	27.4 ± 7.7	24.2 ± 3.1	193	158	PCR-RFLP	CCL17 − 431C/T

CCL = CC chemokine ligand, PCR-RFLP = polymerase chain reaction-restriction fragment length polymorphism, PCR-SSCP = polymerase chain reaction-single stranded conformation polymorphism.

**Table 2:** Allele and genotype information of each gene in each single included study.

Gene	Case					Control					HWE
CCL5											
−403G/A	AA	AG	GG	A	G	AA	AG	GG	A	G	
Kozma	4	38	86	46	210	8	84	211	100	506	0.99
Nickel	6	72	110	84	292	0	28	70	28	168	0.26
Tanaka	44	184	161	272	506	22	67	88	111	243	0.27
−28C/G	GG	GC	CC	G	C	GG	GC	CC	G	C	
Kozma	0	8	120	8	248	0	19	284	19	587	0.85
Tanaka	8	153	228	169	609	7	40	130	54	300	0.25
CCL11											
−426C/T	CC	CT	TT	C	T	CC	CT	TT	C	T	
Rigoli	83	45	2	211	49	50	15	0	115	15	0.57
Tsunemi	118	21	1	257	23	121	16	3	258	22	0.04
−384A/G	AA	AG	GG	A	G	AA	AG	GG	A	G	
Rigoli	81	49	0	211	49	40	25	0	105	25	0.26
Tsunemi	69	58	13	196	84	70	62	8	202	78	0.48
−67G/A	GG	GA	AA	G	A	GG	GA	AA	G	A	
Rigoli	81	48	1	210	50	40	25	0	105	25	0.16
Tsunemi	118	19	3	255	25	110	30	0	250	30	0.36
CCL17											
−431C/T	CC	CT	TT	C	T	CC	CT	TT	C	T	
Sekiya	47	81	20	175	121	61	79	18	201	115	0.60
Tsunemi	61	106	26	193	228	61	79	18	201	115	0.60

CCL = CC chemokine ligand, HWE = Hardy Weinberg Equilibrium.

Genotype frequencies were estimated based on reported allele frequencies, assuming HWE among cases and controls.

### 3.2. Association between CCL5 polymorphisms (−403G/A, −28C/G) and AD risk

**Figure 2.**
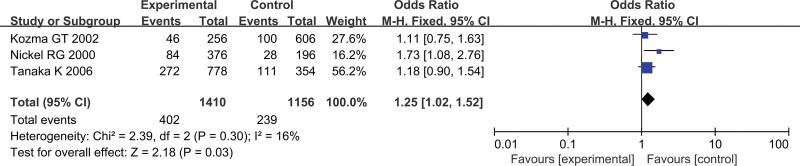
Association between the CCL5 − 403G/A polymorphism and AD risk in the allelic model. AD = atopic dermatitis, CCL = CC chemokine ligand.

**Figure 3.**
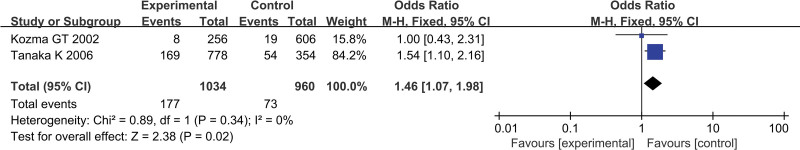
Association between the CCL5 − 28C/G polymorphism and AD risk in the allelic model. AD = atopic dermatitis, CCL = CC chemokine ligand.

Three studies concerned the CCL5 − 403G/A variant, including 705 AD patients and 578 controls. Our meta-analysis revealed a significant association between the CCL5 − 403G/A polymorphism and AD for the allelic model (A vs G: OR = 1.25, 95% CI = 1.02–1.52, *P = *.03) (Fig. 2), heterozygous model (AG vs GG: OR = 1.40, 95% CI = 1.08–1.80, *P = *.01) and dominant model (AA + AG vs GG: OR = 1.38, 95% CI = 1.08–1.76, *P = *.01) in a fixed-effect model as shown in Table 3. However, this significant correlation was not found in other genetic models (AA vs GG: OR = 1.27, 95% CI = 0.77–2.10, *P = *.35; AA vs AG + GG: OR = 1.06, 95% CI = 0.65–1.72, *P = *.81) in a fixed-effect model. For the CCL5 − 28C/G polymorphism, only 2 studies were included. Our result showed that the − 28C/G polymorphism was related to AD susceptibility in the genetic models (G vs C: OR = 1.46, 95% CI = 1.07–1.98, *P = *.02; GG + GC vs CC: OR = 1.74, 95% CI = 1.23–2.47, *P = *.002), except the heterozygous model (GC vs CC: OR = 1.62, 95% CI = 0.77–3.41, *P = *.21) (Fig. 3). Because the GG genotype counts of the experimental group and control group were zero in 1 study, the genetic models GG vs CC and GG vs GC + CC could not be calculated.

**Table 3:** Meta-analysis of CCL5, CCL11 and CCL17 polymorphisms with risk of AD.

	Test for association		Test for heterogeneity		
Genetic model	OR (95% CI)	*P*	*P*H	*I^2^*	Model
CCL5 − 403G/A					
A vs G	1.25 (1.02, 1.52)	.03	0.30	16%	F
AA vs GG	1.27 (0.77, 2.10)	.35	0.39	0%	F
AG vs GG	1.40 (1.08, 1.80)	.01	0.48	0%	F
AA + AG vs GG	1.38 (1.08, 1.76)	.01	0.42	0%	F
AA vs AG + GG	1.06 (0.65, 1.72)	.81	0.36	2%	F
CCL5 − 28C/G					
G vs C	1.46 (1.07, 1.98)	.02	0.34	0%	F
GC vs CC	1.62 (0.77, 3.41)	.21	0.10	62%	R
GG + GC vs CC	1.74 (1.23, 2.47)	.002	0.16	49%	F
CCL11 − 426C/T					
C vs T	0.73 (0.47, 1.12)	.15	0.23	30%	F
CC vs TT	1.24 (0.26, 5.88)	.79	0.26	21%	F
TC vs TT	1.90 (0.37, 9.80)	.44	0.34	0%	F
CC + TC vs TT	1.37 (0.29, 6.60)	.69	0.29	11%	F
CC vs TC + TT	0.67 (0.42, 1.07)	.09	0.34	0%	F
CCL11 − 384A/G					
A vs G	0.94 (0.69, 1.27)	.68	0.70	0%	F
AA vs GA + GG	0.99 (0.69, 1.44)	.97	0.88	0%	F
CCL11 − 67G/A					
A vs G	0.71 (0.48, 1.04)	.08	0.48	0%	F
AA vs GG	3.69 (0.44, 30.96)	.23	0.50	0%	F
GA vs GG	0.75 (0.49, 1.16)	.20	0.29	10%	F
AA + GA vs GG	0.81 (0.53, 1.25)	.34	0.43	0%	F
AA vs GA + GG	3.92 (0.47, 32.66)	.21	0.48	0%	F
CCL17 − 431C/T					
C vs T	0.83 (0.66, 1.03)	.09	0.99	0%	F
CC vs TT	0.69 (0.42, 1.15)	.16	1.00	0%	F
TC vs TT	0.93 (0.57, 1.51)	.76	0.99	0%	F
CC + TC vs TT	0.82 (0.52, 1.31)	.42	0.99	0%	F
CC vs TC + TT	0.74 (0.53, 1.02)	.06	0.98	0%	F

*I^2^*, <25%, no heterogeneity; 25%–50%, modest heterogeneity; >50%, high heterogeneity.

AD = atopic dermatitis, CCL = CC chemokine ligand, CI = confidence interval, F = the fixed-effect model, OR = odds ratio, *P*H = *P*-value of heterogeneity, R = the random-effect model.

### 3.3. Association between CCL11 polymorphisms (−426C/T, −384A/G, −67G/A) and AD risk

**Figure 4.**
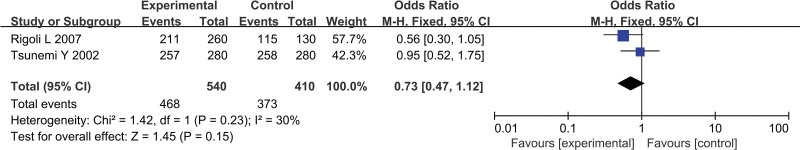
Association between the CCL11 − 426C/T polymorphism and AD risk in the allelic model. AD = atopic dermatitis, CCL = CC chemokine ligand.

**Figure 5.**
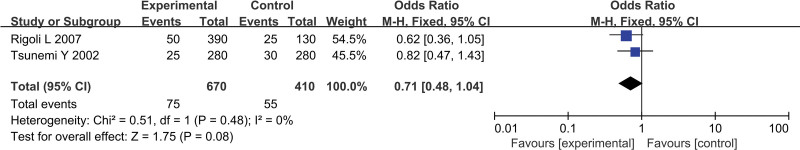
Association between the CCL11 − 67G/A polymorphism and AD risk in the allelic model. AD = atopic dermatitis, CCL = CC chemokine ligand.

**Figure 6.**
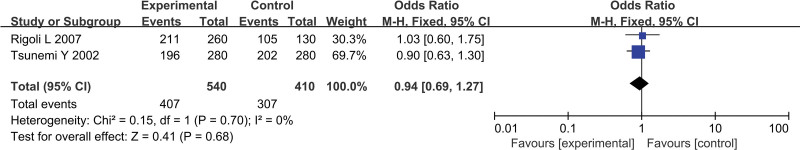
Association between the CCL11 − 384A/G polymorphism and AD risk in the allelic model. AD = atopic dermatitis, CCL = CC chemokine ligand.

The summary ORs and tests for heterogeneity were shown in Table 3. There were 2 studies containing 270 patients and 205 controls. No heterogeneity was found between the studies, and the fixed-effect model was employed to synthesize these data. Even though the frequency of the T allele was slightly higher in AD cases than that in controls (13% vs 9%), no significant association was found between the CCL11 − 426C/T polymorphism and AD risk under 5 genetic models (C vs T: OR = 0.73, 95% CI = 0.47–1.12, *P = *.15; CC vs TT: OR = 1.24, 95% CI = 0.26–5.88, *P = *.79; TC vs TT: OR = 1.90, 95% CI = 0.37–9.80, *P = *.44; CC + TC vs TT: OR = 1.37, 95% CI = 0.29–6.60, *P = *.69; CC vs TC + TT: OR = 0.67, 95% CI = 0.42–1.07, *P = *.09) (Fig. 4). For the CCL11 − 67G/A polymorphism, only 2 studies were included. We found that the − 67G/A polymorphism was not related to AD susceptibility in any genetic models (A vs G: OR = 0.71, 95% CI = 0.48–1.04, *P = *.08; AA vs GG: OR = 3.69, 95% CI = 0.44–30.96, *P = *.23; GA vs GG: OR = 0.75, 95% CI = 0.49–1.16, *P = *.2; AA + GA vs GG: OR = 0.81, 95% CI = 0.53–1.25, *P = *.34; AA vs GA + GG: OR = 3.92, 95% CI = 0.47–32.66, *P = *.21) (Fig. 5). For the CCL11 − 384A/G polymorphism, only 2 studies were included. Our evaluation results showed that the − 384A/G polymorphism was not related to AD susceptibility in the genetic models (A vs G: OR = 0.94, 95% CI = 0.69–1.27, *P = *.68; AA vs GA + GG: OR = 0.99, 95% CI = 0.69–1.44, *P = *.97) (Fig. 6). As above, we could not calculate the genetic model AA vs GG, GA vs GG and GG vs AA + GA, because the GG genotype counts were zero in the experimental and control groups in 1 study.

### 3.4. Association between CCL17 polymorphism (−431C/T) and AD risk

**Figure 7.**
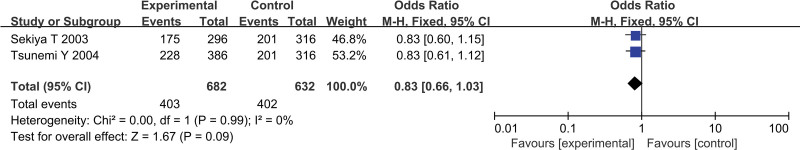
Association between the CCL17 − 431C/T polymorphism and AD risk in the allelic model. AD = atopic dermatitis, CCL = CC chemokine ligand.

For the CCL17 − 431C/T polymorphism, 2 studies were included (341 patients and 316 controls). We did not find a significant correlation between the CCL17 − 431C/T polymorphism and AD risk (C vs T: OR = 0.83, 95% CI = 0.66–1.03, *P = *.09; CC vs TT: OR = 0.69, 95% CI = 0.42–1.15, *P = *.16; TC vs TT: OR = 0.93, 95% CI = 0.57–1.51, *P = *.76; CC + TC vs TT: OR = 0.82, 95% CI = 0.52–1.31, *P = *.42; CC vs TC + TT: OR = 0.74, 95% CI = 0.53–1.02, *P = *.06) (Fig. 7).

### 3.5. Sensitivity analysis and publication bias

Each study in each comparison model was deleted every time to estimate whether the single article affected the overall ORs. Our results showed that the pooled ORs were not significantly changed. Because the number of included studies was < 10, we did not assess the publication bias.

## 4. Discussion

In this meta-analysis, we screened out 7 relevant studies. Our results showed that the A allele of CCL5 − 403G/A in the allelic model, the AG genotype of CCL5 − 403G/A in the heterozygous model, the AA + AG genotype of CCL5 − 403G/A in the dominant model, the G allele of CCL5 − 28C/G in the allelic model and the GG + GC genotype of CCL5 − 28C/G in the dominant model were significantly associated with an increased risk of AD. This significant association was not found either in other genotypes of the CCL5 − 403G/A and CCL5 − 28C/G polymorphisms, or in all alleles and genotypes of the CCL11 − 426C/T, CCL11 − 384A/G, CCL11 − 67G/A and CCL17 − 431C/T polymorphisms. This is the first meta-analysis concerning these 3 gene polymorphisms in AD risk.

CCL5 is a migration factor for eosinophils which is involved in the recirculation of memory T cells. With the help of the cytokines IL-2 and interferon-gamma (IFN-γ) released by T cells, CCL5 can induce the proliferation and activation of certain natural killer cells to form CC chemokine-activated killer cells. CCL5 can induce immunoglobulin E (IgE) production, which is linked to the pathophysiology of anaphylaxis and other acute allergic reactions.^[28]^ Elevated IgE responses may reflect increased responses of Th2 cytokines with a concomitant decrease in IFN-γ production in patients with AD.^[29]^ Some pathogens such as *Malassezia*, might exert their role by specifically binding to IgE. When the antigen-presenting cells present the antigens, they induce the differentiation of Th0 cells into Th2 cells. Then Th2 cells secrete chemokines like CCL5, which can promote B cells to produce a large number of specific IgE.^[30]^ Gluck et al^[31]^ and Kaburagi et al^[32]^ showed that the serum CCL5 level in the AD group was higher than that in the healthy control group. Yamada et al^[33]^ showed that CCL5 mRNA was more expressed in the skin of patients with AD. Giustizieri et al^[34]^ demonstrated that keratinocytes from AD patients have distinct chemokine production properties in response to T cell-derived cytokines. However, the meta-analysis of Wen et al^[35]^ showed that CCL5 − 403G/A and − 28C/G were not associated with AD. In our study, we found that the A allele of CCL5 − 403G/A in the allelic model, the AG genotype of CCL5 − 403G/A in the heterozygous model, the AA + AG genotype of CCL5 − 403G/A in the dominant model, the G allele of CCL5 − 28C/G in the allelic model, and the GG + GC genotype of CCL5 − 28C/G in the dominant model were significantly associated with an increased risk of AD.

CCL11 is a powerful chemotactic and activator that can act on eosinophils, basophils and Th2 lymphocytes via the chemokine receptor CC chemokine receptor 3 (CCR3) in the serum. The CCL11 promoter contains common binding sites overlapping transcription factors, nuclear factor-kappa B and signal transducer and activator of transcription 6, which mediate tumor necrosis factor-α and IL-4 responses.^[36,37]^ Antigen-induced expression of CCL11 by endothelial cells and the adhesion and transmigration of eosinophils from microvessels to tissues would be controlled in part by histamine released from degranulated mast cells. Recently, a new therapeutic strategy that targets blocking CCL11 signaling has shown significant improvement in patients with moderate-to-severe AD.^[38]^ Immunoreactivity and transcripts of CCL11 were significantly increased in lesional skin from AD patients, but not in nonatopic controls.^[39]^ Owczarek et al^[40]^ showed that the average expression level of CCL11 gene in skin changes of AD patients was higher than that of unaffected skin. Hossny et al^[41]^ showed that CCL11 was elevated in the plasma of patients with infantile AD. Jahnz-Rozyk et al^[42]^ showed that CCL11 levels in the sera from patients with AD were significantly elevated compared to those from healthy volunteers. It may be a useful inflammatory marker that correlates with the extent component of AD in particular, and differentiates mild disease from more severe disease when used for assessing AD severity in young children. Previous studies have suggested that CCL11 gene polymorphisms might be involved in the development of AD by contributing to a functional dysregulation of CCL11 production in vivo.^[27,43]^ In our study, we found that the CC genotypes of CCL11 − 426C/T, CCL11 − 384A/G and CCL11 − 67G/A were not associated with an increased risk of AD in any of the models.

CCL17/thymus and activation-regulated chemokine (TRAC), a chemoattractant of TH2 cells, has some significant evidences for robust correlation with AD clinical severity, at both baseline and during therapy.^[44]^ CCL17 binds specifically and exclusively to CCR4, which is selectively expressed on Th2 cells and elicits selective and potent migratory responses in Th2 cells in vitro.^[45]^ CCL17 acts as a downstream mediator of granulocyte-macrophage colony-stimulating factor in monocytes/macrophages as well as in models, and granulocyte-macrophage colony-stimulating factor can induce CCL17 to mediate inflammation via IFN regulatory factor 4 which is regulated by Jumonji domain-containing protein-3.^[46]^ CCL17 sensitizes DCs for CCR7- and CXCR4-dependent migration to lymph node-associated homeostatic chemokines under inflammatory conditions and thus plays an important role in cutaneous DCs migration.^[47]^ AD patients had higher serum CCL17 level and higher CCL17 expression on peripheral blood mononuclear cells compared to normal subjects.^[48,49]^ The contents of TRAC in platelets and the plasma levels of TRAC and macrophage-derived chemokine in patients with AD were elevated.^[50]^ Esenboga et al^[51]^ showed that in children with AD, the severity of disease was positively correlated with the level of CCL17. The serum CCL17 level was also strongly correlated with disease activity of AD.^[52,53]^ In our study, we did not find a significant association between the CCL17 − 431C/T variant and AD risk.

However, several limitations were presented in this meta-analysis. Firstly, AD is a very heterogeneous disease, the severity of disease was not reported in most included studies, which might restrict the accuracy of our results. Secondly, the number of included studies for some gene mutations was small. Thirdly, the inclusion of adult cases with childhood-and adult-onset AD or unknown AD onset and cases with various concomitant atopic diseases added to the heterogeneity of the phenotype studied in this meta-analysis. Fourthly, there was slight publication bias in our meta-analysis. Lastly, the contributions of haplotype and interaction effects involving the polymorphism to AD require further studies.

## 5. Conclusions

In conclusion, our results suggest that the A allele of CCL5 − 403G/A in the allelic model, the AG genotype of CCL5 − 403G/A in the heterozygous model, the AA + AG genotype of CCL5 − 403G/A in the dominant model, the G allele of CCL5 − 28C/G in the allelic model and the GG + GC genotype of CCL5 − 28C/G in the dominant model might be significantly associated with an increased risk of AD. More large-scale studies should be carried out to confirm or refute the association between chemokines gene polymorphisms and the risk of AD.

## Author contributions

**Conceptualization:** Lu Mao, Chenghui Zou.

**Data curation:** Lu Mao, Chenghui Zou.

**Formal analysis:** Lu Mao, Chenghui Zou, Mao Li, Dan He, Yujie Han.

**Investigation:** Dan He, Yujie Han.

**Methodology:** Mao Li, Min Liu.

**Project administration:** Min Liu.

**Software:** Lu Mao, Chenghui Zou, Mao Li, Dan He, Yujie Han.

**Supervision:** Min Liu.

**Validation:** Min Liu.

**Visualization:** Wen Zhang.

**Writing – original draft:** Lu Mao, Chenghui Zou, Wen Zhang.

**Writing – review & editing:** Min Liu.

## Appendix

### Details of the literature search procedure and strategy

Appendix: Search Form

Chinese databases:

Search keywords such as atopic dermatitis, chemotactic factors, including atopic dermatitis, atopic eczema, chemotactic factors, chemokine ligand 5, chemokine ligand 11, chemokine ligand 17, etc. Similar keywords are connected with “or,” and the different keywords are connected with “and” to get the search result.

1. Search strategy used in CNKI.

**Table:**

Database	Keyword range	No	Search items
CNKI	主题	1	(特应性皮炎 or 特应性湿疹 or 异位性湿疹 or 异位性皮炎 or 遗传过敏性湿疹 or 遗传过敏性皮炎) AND (趋化因子类 or 趋化细胞因子 or CC类趋化因子 or 趋化因子配体5 or 趋化因子配体11 or 趋化因子配体17 or 嗜酸性粒细胞趋化因子 or 胸腺活化调节趋化因子)

2. Search strategy used in Wanfang.

**Table:**

Database	Keyword range	No	Search items
Wanfang	题名或关键词	1	(特应性皮炎) or (特应性湿疹) or (异位性湿疹) or (异位性皮炎) or (遗传过敏性湿疹) or (遗传过敏性皮炎)
		2	(趋化因子类) or (趋化细胞因子) or (CC类趋化因子) or (趋化因子配体5) or (趋化因子配体11) or (趋化因子配体17) or (嗜酸性粒细胞趋化因子) or (胸腺活化调节趋化因子)
		3	1 and 2

3. Search strategy used in VIP.

**Table:**

Database	No	Search items
VIP	1	M=(特应性皮炎+特应性湿疹+异位性湿疹+异位性皮炎+遗传过敏性湿疹+遗传过敏性皮炎)
	2	M=(趋化因子类+趋化细胞因子+CC类趋化因子+趋化因子配体5+趋化因子配体11+趋化因子配体17+嗜酸性粒细胞趋化因子+胸腺活化调节趋化因子)
	3	1 and 2

4. Search strategy used in CBM.

**Table:**

Database	Keyword range	No	Search items
CBM	主题：[常用字段]	1	(((((“特应性皮炎”) OR “特应性湿疹”) OR “异位性湿疹”) OR “异位性皮炎”) OR “遗传过敏性湿疹”) OR “遗传过敏性皮炎”)
		2	(((((((((((“趋化因子类”) OR “趋化细胞因子”) OR “ CC类趋化因子”) OR “趋化因子配体5”) OR “趋化因子配体11”) OR “趋化因子配体17”) OR “嗜酸性粒细胞趋化因子”) OR “胸腺活化调节趋化因子”)
		3	1 and 2

English databases:

First, enter the Pubmed-MeSH database and search for entry terms for all keywords. Second, search for each keyword and its entry term separately, connecting them with an “or.” Finally, connect the search results with “and” to get the results.

5. Search strategy used in Pubmed.

**Table:**

No	Search items
1	Dermatitis, Atopic[mh] or Atopic Dermatitides[af] or Atopic Dermatitis[af] or Dermatitides, Atopic[af] or Neurodermatitis, Atopic[af] or Atopic Neurodermatitides[af] or Atopic Neurodermatitis[af] or Neurodermatitides, Atopic[af] or Neurodermatitis, Disseminated[af] or Disseminated Neurodermatitides[af] or Disseminated Neurodermatitis[af] or Neurodermatitides, Disseminated[af] or Eczema, Atopic[af] or Atopic Eczema[af] or Eczema, Infantile[af] or Infantile Eczema[af]
2	Chemotactic Factors[mh] or Chemotactic cytokines[af] or Chemokine[af] or Chemotaxis[af] or Chemotaxis, Leukocyte [af]
3	Chemokine ligand 5[mh] or RANTES[af] or C-Cmotif Chemokine 5[af] or regulated upon activation normal T cell expressed and secreted[af]
4	Chemokine ligand 11[mh] or Eotaxin[af] or eotaxin-1[af] or C-C motif chemokine 11[af] or chemokine(C-C motif)ligand 11[af] or chemokine C-C motif chemokine 11[af] or MGC22554[af] or SCYA11[af]
5	Chemokine ligand 17[mh] or TARC[af] or R 41400[af] or R41400[af] or R41,400[af] or Nizoral[af]
6	3 or 4 or 5
7	1 and 2 and 6

6. Search strategy used in Embase.

**Table:**

No	Search items
1	“dermatitis, atopic”/exp or “dermatitis, atopic” or dermatitis, and atopicor “atopic dermatitides” or atopic and dermatitidesor “atopic dermatitis”/exp or “atopic dermatitis” or atopic and “dermatitis”/exp or dermatitisor “dermatitides, atopic” or dermatitides, and atopicor “neurodermatitis, atopic” or neurodermatitis, and atopicor “atopic neurodermatitides” or atopic and neurodermatitidesor “atopic neurodermatitis” or atopic and “neurodermatitis”/exp or neurodermatitisor “neurodermatitides, atopic” or neurodermatitides, and atopicor “neurodermatitis, disseminated” or neurodermatitis, and disseminatedor “disseminated neurodermatitides” or disseminated and neurodermatitidesor “disseminated neurodermatitis” or disseminated and “neurodermatitis”/exp or neurodermatitisor “neurodermatitides, disseminated” or neurodermatitides, and disseminatedor “eczema, atopic” or eczema, and atopicor “atopic eczema”/exp or “atopic eczema” or atopic and “eczema”/exp or eczemaor “eczema, infantile”/exp or “eczema, infantile” or eczema, and infantileor “infantile eczema”/exp or “infantile eczema” or infantile and “eczema”/exp or eczema
2	“chemotactic factors”:ab,ti or “chemotactic cytokines”:ab,ti or chemokines:ab,ti or ‘ chemotaxis “:ab,ti or” chemotaxis, Leukocyte ‘:ab,ti
3	“chemokine ligand 5”: ab,ti or rantes:ab,ti or ‘ c-c motif chemokine 5 ‘:ab,ti or ‘regulated upon activation normal t cell expressed and secreted ‘:ab,ti
4	“chemokine ligand 11”: ab,ti or eotaxin:ab,ti or eotaxin-1:ab,ti or “c-c motif chemokine 11”:ab,ti or “chemokine (c-c motif)ligand 11”:ab,ti or mgc22554:ab,ti or scya11:ab,ti or “chemokine c-c motif chemokine 11”:ab,ti
5	“chemokine ligand 17”: ab,ti or tack:ab,ti or “c-c motif chemokine 17”:ab,ti or “cc chemokine tarc”:ab,ti or scya 17:ab,ti or “ccl17 elisa kit”:ab,ti or “tarc from mouse”:ab,ti or “thymus activation regulated chemokine”:ab,ti
6	2 or 3 or 4 or 5
7	1 and 6

7. Search strategy used in Cochrane Library.

**Table:**

No	Search items
1	Dermatitis, Atopic or Atopic Dermatitides or Atopic Dermatitis or Dermatitides, Atopic or Neurodermatitis, Atopic or Atopic Neurodermatitides or Atopic Neurodermatitis or Neurodermatitides, Atopic or Neurodermatitis, Disseminated or Disseminated Neurodermatitides or Disseminated Neurodermatitis or Neurodermatitides, Disseminated or Eczema, Atopic or Atopic Eczema or Eczema, Infantile or Infantile Eczema
2	Chemotactic Factors or Chemotactic Cytokines or Chemokines or Chemotaxis or Chemotaxis, Leukocyte
3	1 and 2
4	MeSH descriptor [Chemokine Ligand 5] explode all trees
5	MeSH descriptor [Chemokine Ligand 11] explode all trees
6	MeSH descriptor [Chemokine Ligand 17] explode all trees
7	4 or 5 or 6
8	3 and 7
